# A Study on Rare Earth-Modified Co-Based Composite Powder and Its Effects on the Microstructure and Properties of Cemented Carbide

**DOI:** 10.3390/ma18071494

**Published:** 2025-03-27

**Authors:** Zhiyong Li, Azman Jalar, Norinsan Kamil Othman

**Affiliations:** 1 Faculty of Science and Technology, Universiti Kebangsaan Malaysia, Bangi 43600, Malaysia; insan@ukm.edu.my; 2 School of Intelligent Manufacturing, Guangdong Technology College, Zhaoqing 526100, China

**Keywords:** cemented carbide, rare earth, co-based composite powder, phase, microstructure, properties

## Abstract

A Co-based composite powder, doped with rare earth Y, was crafted through a series of processes involving spray-drying, calcination, and low-temperature reduction. This powder was then blended with tungsten carbide (WC) powder and subjected to ball-milling. The resultant mixture was consolidated into a robust Y-doped WC-Co cemented carbide via the process of spark plasma sintering (SPS). The outcomes demonstrate that incorporating rare earth Y into Co powder to form a Co-Y_2_O_3_ composite powder via an innovative spray-drying, calcination, and low-temperature reduction process ensures uniform distribution of Y in the Co matrix. This uniform distribution refines the alloy’s grain structure during subsequent sintering, leading to enhanced performance. Within a specific range, increasing the Y content improves the overall alloy properties. It is notable that at a Y content of 1.5%, the alloy’s properties reach a state of stability, characterized by a density of 98.91%, a maximum hardness of 2120 Hv_30_, and a fracture toughness of 8.24 MPa·m^1/2^. The novel Y incorporation method has been shown to enhance the strength of the binder phase, impede the growth of WC grains, and thereby lead to a substantial improvement in the overall performance of the cemented carbide.

## 1. Introduction

Cemented carbide is a material that has found wide application in a variety of fields, including machining, petroleum/mining, mold processing, and the fabrication of structural wear-resistant parts. The reasons for this are threefold: namely, its high strength, high hardness, and good toughness [1,2,3]. Studies [4,5] have shown that the performance of cemented carbide is primarily influenced by its microstructure and composition, including factors such as the grain size of refractory metal carbides and the composition of the Co-binding phase.

WC is the most commonly used carbide raw material for cemented carbide due to its exceptional hardness and wear resistance, while Co is the most commonly used binding phase element in cemented carbide, owing to the extremely low wetting angle between it and WC, which significantly enhances the toughness and strength of the alloy [6]. However, the mechanical properties of WC-Co cemented carbides are closely related to the grain size of the WC matrix. It has been established that refining the WC grain size can enhance hardness and strength, thus improving the performance of the alloy [7]. To date, the most effective method of refining WC grains is the addition of grain-refining additives. Research on additives for cemented carbides primarily includes carbide additives (e.g., VC, Cr_3_C_2_, and TaC), carbon nanotubes, and rare earth additives. The addition of VC has been shown to inhibit the growth of WC grains, thereby refining the grains and enhancing the hardness of the alloy. However, VC has also been observed to increase the porosity of the alloy, resulting in elevated brittleness and reduced strength [8]. Furthermore, VC has been found to have a minimal effect on the alloy’s corrosion resistance [9,10]. In a related study, Yi et al. [11] incorporated Cr_3_C_2_ into WC-Co hard alloys, observing that Cr_3_C_2_ effectively curtailed WC grain growth, thereby enhancing alloy hardness and corrosion resistance. However, this process concomitantly diminished alloy strength. The addition of carbon nanotubes to cemented carbides has been shown to enhance their performance; however, the poor dispersion and tendency to agglomerate within the carbide matrix have resulted in limited research on the effects of additives such as carbon nanotubes on the corrosion resistance of WC-Co cemented carbides [12,13].

Rare earth elements are known to exhibit high chemical reactivity; the current focus of research in this area is on the types and concentrations of rare earth additives [14,15]. Deng et al. [16] demonstrated that doping rare earth oxide CeO_2_ into WC-Co cemented carbides significantly enhances alloy performance. In a similar vein, Jiang et al. [17] investigated the distribution of La_2_O_3_ in WC-Co cemented carbides, finding that La_2_O_3_ effectively improves the wettability at the WC/Co interface, suppresses continuous WC grain growth, and strengthens grain and phase boundaries, thereby enhancing the alloy’s strength and toughness.

Moreover, the manner in which rare earth elements are incorporated exerts a significant influence on the properties of the resultant alloys. The prevailing research approach, which is predominantly focused on the utilization of ball milling as a means to introduce rare earth additives into cemented carbides, is accompanied by the observation that this method tends to result in the concentration of additives at the WC/WC and WC/Co interfaces. This phenomenon leads to an uneven distribution of the additives and consequent reduction in the performance of the alloys [18]. It is evident that the method of additive incorporation is closely related to its distribution uniformity within the alloy. Conventional solid-phase addition techniques, such as ball milling, exhibit limited effectiveness in enhancing additive uniformity and are associated with relatively high energy consumption. Therefore, developing an alternative approach that significantly improves the uniform distribution of additives is an urgent necessity.

This study proposes a methodology involving the combination of a spray drying process with a calcination reduction process. The purpose of this combination is to introduce Y_2_O_3_ in solution form into Co powder. This approach allows for the molecular-level distribution of rare earth elements Y and Co within the alloy, thereby enhancing its performance. The study employs a systematic approach, varying the amount of Y_2_O_3_ added to the WC-Co cemented carbide samples, to investigate the impact of the rare earth element Y on the microstructure, mechanical properties, and corrosion resistance of the resultant alloys. This comprehensive investigation provides a robust theoretical foundation for the development of WC-Co alloys that exhibit superior comprehensive properties.

## 2. Experimental Materials and Methods

### 2.1. Materials and Fabrication Method

The raw materials used in this study were WC powder (average particle size 0.5 μm, purity 99.5%, purchased from Jiangxi Yaosheng Tungsten Industry Co., Ltd., in Ganzhou, China), (CH_3_COO)_2_Co·4H_2_O (99.5%, purchased from Shanghai Aladdin Biochemical Technology Co., Ltd., in Shanghai, China), and Y(C_2_H_3_O_2_)_3_·4H_2_O (99.5%, purchased from Shanghai Aladdin Biochemical Technology Co., Ltd., in Shanghai, China). The experimental equipment primarily consisted of a spray dryer (Wuxi Nengda Drying Equipment Co., Ltd., Wuxi, China), a reduction furnace (Hunan Dingli Technology Co., Ltd., Changsha, China), a planetary ball mill (Nanjing Nanda Instrument Co., Ltd., Nanjing, China), and a spark plasma sintering (SPS) furnace (Shanghai Chenhua Technology Co., Ltd., Shanghai, China).

Firstly, the raw powder materials should be weighed precisely according to the ratios in Table 1, and the weighed WC powder should be sealed for subsequent use. Next, the (CH_3_COO)_2_Co·4H_2_O and Y(C_2_H_3_O_2_)_3_·4H_2_O powders should be gradually added into a beaker containing a suitable amount of deionized water, and the solution should be stirred to ensure complete dissolution of the materials and achieve a homogeneous mixture. The mixed solution should then be subjected to ultrasonication for a period of five minutes, a process that effectively promotes the uniform distribution of the Co and Y elements at the molecular level, thus ensuring the uniformity of the precursor material.

Following the ultrasonic dispersion process, the resultant solution was transferred into the feeding system of the spray dryer. The spray dryer then atomizes the solution into minute droplets via a high-pressure nozzle, causing rapid evaporation of water in the hot air and subsequent solidification into fine powder particles to yield precursor powder containing uniformly dispersed Co and Y elements. The obtained precursor powder samples were designated as A1, A2, A3, A4, and A5. Subsequently, the precursor powder was transferred to a high-temperature furnace for calcination, with the objective of forming a stable crystal structure. The calcined powder samples were labeled as B1, B2, B3, B4, and B5. Thereafter, a reduction reaction was performed in a hydrogen atmosphere, with the objective of removing the oxygen from the oxide. This process yielded a pure Co-Y_2_O_3_ composite powder. The reduced powder samples were labeled as C1, C2, C3, C4, and C5.

Following this, the WC powder and the reduced Co-Y_2_O_3_ composite powder, previously weighed, were placed into the carbide alloy lining ball grinding tank for planet grinding. The grinding balls were carbide balls with a 6 mm diameter, the ball material ratio was 5:1, the ball grinding time was 10 h, and the ball grinding speed was 160 r/min. The ball grinding slurry was then subjected to heating in a vacuum drying box at a temperature of 70 °C for a duration of 4 h. Following this, the resultant powder mixture was cooled and removed.

Finally, the mixture of dried uniform powder was weighed (20 g) and filled into a graphite mold of 20 mm. The mold was placed in the SPS furnace and heated to 1280 °C at a rate of 100 °C/min, with a pressure of 50 MPa applied, and held for 10 min to ensure sufficient densification and alloying of the powder. Based on previous experiments conducted by researchers and our multiple trials, these processing parameters have been found to ensure sufficient densification while preventing excessive grain growth and minimizing the formation of structural defects [19]. This process yielded WC-6Co cemented carbides with varying Y content, as detailed in Table 1 and labeled as Sample 1, Sample 2, Sample 3, Sample 4, and Sample 5.

### 2.2. Characterization

The prepared alloy was initially stripped of the graphite paper on its surface and then subjected to a series of diamond grinding discs with grits of 100, 400, 800, 1000, and 2000, in that order. The surface was then polished with a polishing cloth. Subsequently, the surface was etched with an etchant for 30 s, after which it was rinsed with deionized water and dried thoroughly. The etchant was prepared by mixing 5 wt.% KOH solution and 5 wt.% K_3_Fe(CN)_6_ solution in deionized water.

The morphology of the powder and the alloys was observed using a field-emission scanning electron microscope (FEI Corporation, Hillsboro, OR, USA). The energy spectrum was detected using an X-Flash 5030 energy spectrometer (Bruker Company, Billerica, MA, USA). The mean grain size of WC was measured using the linear intercept method based on SEM images. The actual density of the alloy was measured using the drainage method and calculated using the following formula:

(1) $$\rho =\frac{m}{V_{drain}}$$

where ρ represents density, m represents the sample mass, and V_drain_ represents the volume of displaced water.

And compared with the theoretical density, the relative density is obtained as follows:

(2) $$RD=\frac{\rho_{actual}}{\rho_{theory}}\times 100\%$$

where RD donates the relative density, ρ_actual_ denotes the actual density, and V_theory_ donates the theoretical density.

Determination of the powder and alloy phases was conducted using an Empyream X-ray diffractometer, while the alloy microhardness was measured using a Vickers hardness tester, with the loading force set to 30 kg and a loading time of 15 s. A predetermined load was applied to each point and held for a specified duration, forming a diamond-shaped indentation. The diagonal length of the indentation was measured, and the hardness values of each point were calculated using the Vickers hardness formula. The average value was taken as the microhardness of the sample. The alloy fracture toughness values were calculated using the Palmqvist indentation method, which allows for the assessment of fracture toughness using relatively simple indentation tests, providing valuable insights into the material’s mechanical performance. The indentation test was conducted, and both the diagonal length of the indentation and the crack extension length were measured. The calculation formula is as follows:

(3) $$K_{IC}=\sqrt{\frac{E}{H}}\cdot \frac{P}{C^{\frac{3}{2}}}$$

where C donates the constant, E donates the elastic modulus, H donates the hardness, P donates the load, and c donates the crack half-length [20,21].

## 3. Results

**Figure 1:**
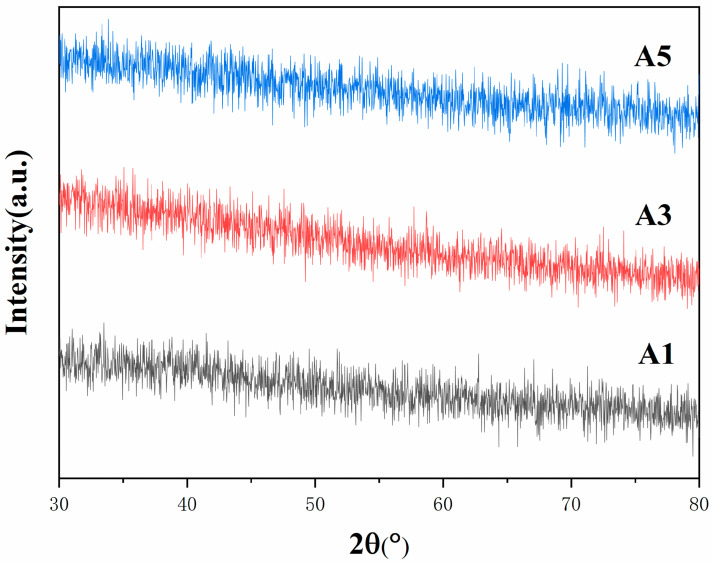
The XRD patterns of precursor powders with various Y contents.

A mixture of Co and Y was prepared with a concentration of 40%, utilizing a centrifugal atomization speed of 12,000 r/min, an air inlet temperature of 220 °C, and an outlet temperature of 140 °C. Five groups of precursor powders with varying Y content (ranging from 0 to 2.0 wt.%) were synthesized. The phase analysis of A1, A3, and A5 is depicted in Figure 1. The XRD patterns directly reflect the effect of the addition of Y on the physical phase of the precursor powder. Although the Y content was varied in the range of 0 to 2.0 wt.%, none of the samples showed obvious crystal characteristic peaks. This clearly indicates that the precursor powder is in an amorphous phase state, i.e., the elements have not yet formed an ordered crystal structure. This outcome suggests that the introduction of Y did not trigger or accelerate the crystallization process of Co-based precursor powder under the current preparation conditions. Furthermore, the XRD peak patterns exhibited a high degree of similarity, and the lack of significant differences due to variations in Y content further confirms that the addition of Y had no substantial effect on the basic phase composition of the precursor powder. Consequently, it can be deduced that within the present concentration range, the Y element may exist in a state of dispersion or in alternative forms, which is insufficient to induce substantial alterations in the phase structure of the precursor powder.

**Figure 2:**
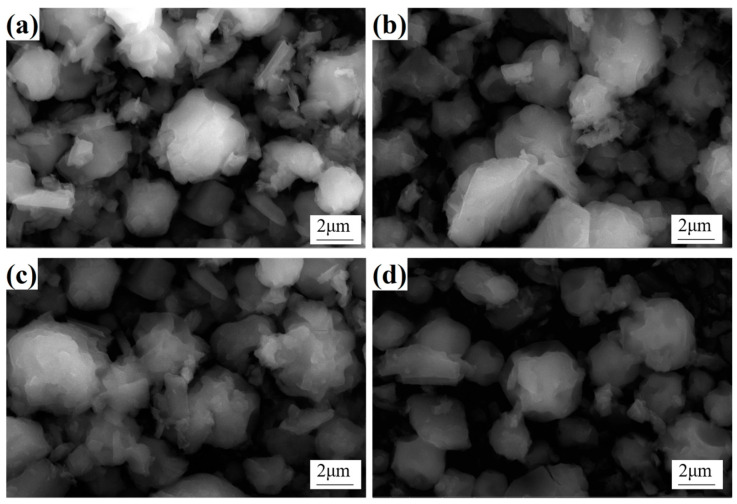
Micromorphology of precursor powders with different Y contents: (**a**) 0; (**b**) 0.5%; (**c**) 1.0%; (**d**) 2.0%.

As illustrated in Figure 2, the morphology of Co precursor powders with different Y contents primarily exhibits a near-spherical structure formed by the aggregation of fine particles. This is attributed to the rapid centrifugal atomization process, where the solution is refined into microdroplets and subsequently undergoes rotational descent within the spray tower. During this process, the surface tension of the droplets causes them to contract, gradually acquiring a near-spherical form. Concurrently, the action of hot air flow accelerates the evaporation of water on the surface of the droplets, resulting in the concentration of solutes such as Y and cobalt salts rising beyond the solubility threshold. This process leads to the discharge of the composite shell containing Y, Co, O, and other elements on the surface of the droplets. As the internal evaporation of water continues, the inside of the droplet gradually crystallizes, finally forming the quasi-spherical structure [22]. It is noteworthy that although the Y content varied in the precursor powder, no obvious micromorphological differences were observed, as shown in Figure 2. This indicates that the addition of Y did not significantly affect the basic morphological structure of the powder in the range of Y content studied. This phenomenon may be attributed to the uniform distribution of the Y element in the powder or its non-dominant role in morphology evolution. In addition, the rapidity and temperature control of the spray conversion process also ensure the consistency of the powder morphology, although the powder still contains a certain amount of adsorbed water and crystalline water, which is determined by the heat absorption during water evaporation and the instantaneous nature of the spray conversion.

**Figure 3:**
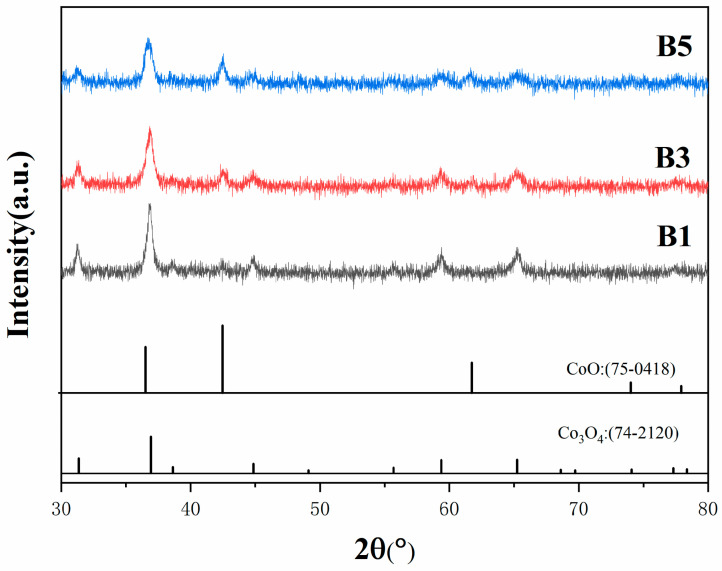
XRD patterns of powders with different Y contents after calcination.

The XRD results of the precursor powders following low-temperature calcination are presented in Figure 3. The calcination process was conducted at a heating rate of 5 °C/min, a calcination temperature of 300 °C, and a holding time of 60 min, in an air atmosphere. The XRD patterns demonstrate that the diffraction peaks of B1, B3, and B5 powders are almost indistinguishable, corresponding to the characteristic peaks of Co_3_O_4_ and CoO. This suggests that the powders were effectively converted into Co_3_O_4_ and CoO following calcination. A detailed analysis of the data revealed that the CoO phase content in the samples increases with the increasing addition of rare earth Y. However, it should be noted that although the Y element was introduced into the precursor, Y was not observed in the XRD results. This phenomenon is most likely due to the relatively low addition of Y, which did not reach the sensitivity threshold for XRD detection. Consequently, while the existence of Y is probable, its contribution to the overall bulk phase structure is not reflected in the XRD pattern. Furthermore, a quantitative analysis of the XRD data was conducted using the RIR method, and the results are presented in Table 2. It can be observed that the samples contain both CoO and Co_3_O_4_ phases, with the weight fraction of CoO gradually increasing from B1 to B5. This indicates that the CoO content in the samples increases with the Y content. In summary, the low-temperature calcination process effectively converted the precursor powder into Co_3_O_4_ and CoO oxides. Furthermore, the CoO content in the samples increased with the addition of Y, and compared to Co_3_O_4_, CoO is more favorable for the subsequent reduction process [23].

**Figure 4:**
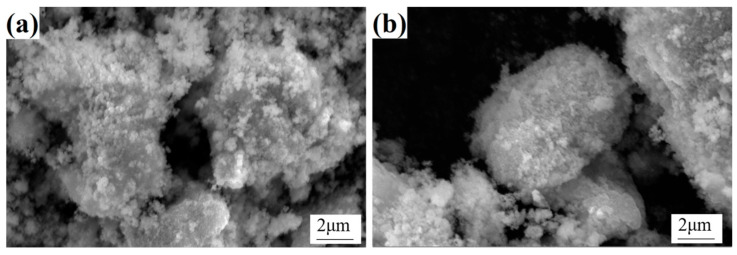
Micromorphology of cobalt oxide powders B3 and B5: (**a**) B3 and (**b**) B5.

In order to investigate the powder morphology before and after calcination, SEM characterization was performed on the calcined oxide powder samples B3 and B5, as shown in Figure 4. A comparison of the precursor powder and the calcined oxide powder revealed that, while the former is characterized by a certain level of similarity in morphology, the latter displays significant changes in surface characteristics. Specifically, the surface of the oxide powder is covered with a substantial number of fine particles, resulting in a substantial increase in the overall surface roughness. This transformation can be ascribed to the volatilization of a substantial quantity of crystalline water originally present in the precursor powder during the calcination process. The removal of crystalline water not only results in the formation of pores within the powder but also promotes the roughening of the powder surface. Concurrently, nucleation and crystal growth occurred in the precursor during calcination. As the temperature rises and the duration extends, the oxide crystals gradually form and grow on the surface and inside of the powder, ultimately resulting in the microscopic morphology depicted in Figure 4. It is noteworthy that despite the variation in the Y content of the precursor powders of A3 and A5, the oxide powders obtained after calcination exhibited no substantial difference in micromorphology. This observation suggests that Y content does not exert a significant influence on the microtissue morphology of the oxide powder. Consequently, it can be deduced that under the prevailing calcination conditions, the Y element predominantly exists as an additive and does not exert a direct influence on the crystal growth and morphology evolution of the oxide.

**Figure 5:**
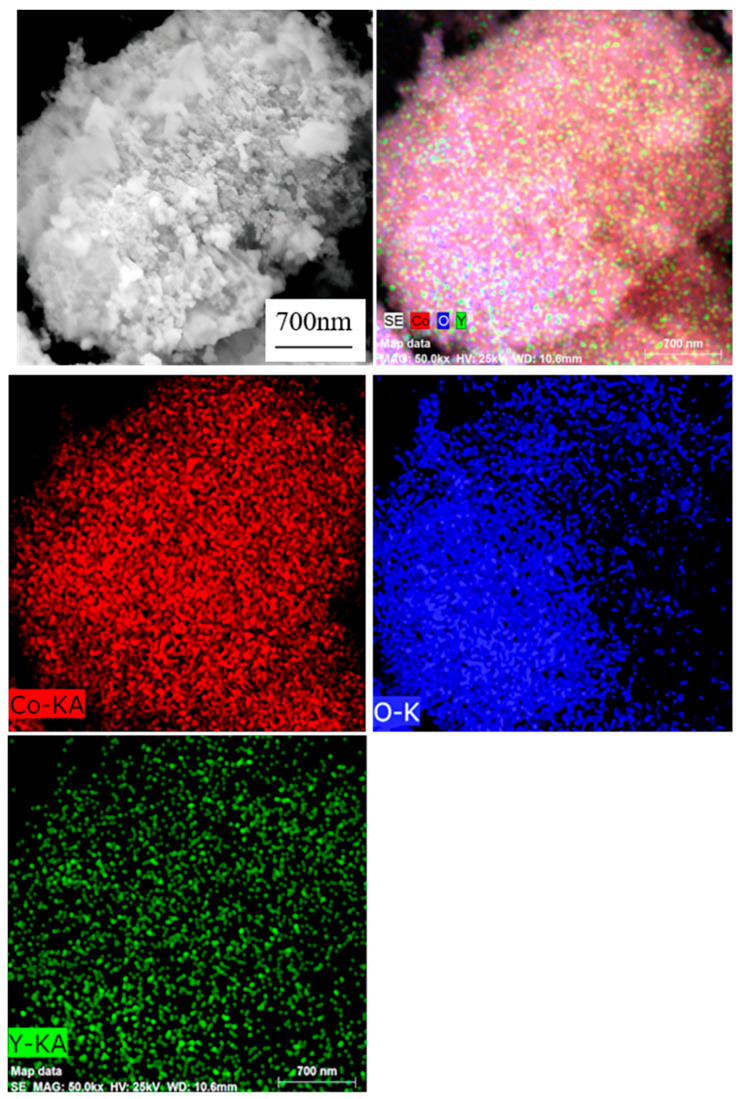

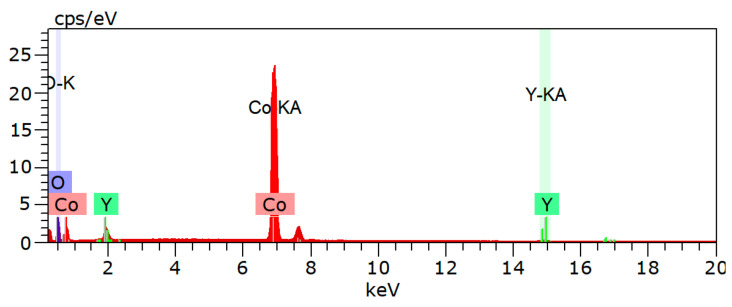
Elemental surface analysis of B5.

As demonstrated in Figure 5, the EDS mapping results of oxide powder B5 clearly indicate that the three elements, Co, Y, and O, exhibit a uniform distribution across the powder surface, with no discernible element aggregation or absence. This observation signifies that the elements have been effectively mixed and distributed and that no extraneous impurities remain present in the powder. The outcomes of this study demonstrate the efficacy of the spray process in introducing the Y element into the precursor powder and the calcination process in preserving the uniform distribution of elements. Consequently, it can be concluded that the oxide powder exhibits a high degree of purity and consistency in its elemental composition, thereby providing a reliable material foundation for subsequent research and applications.

**Figure 6:**
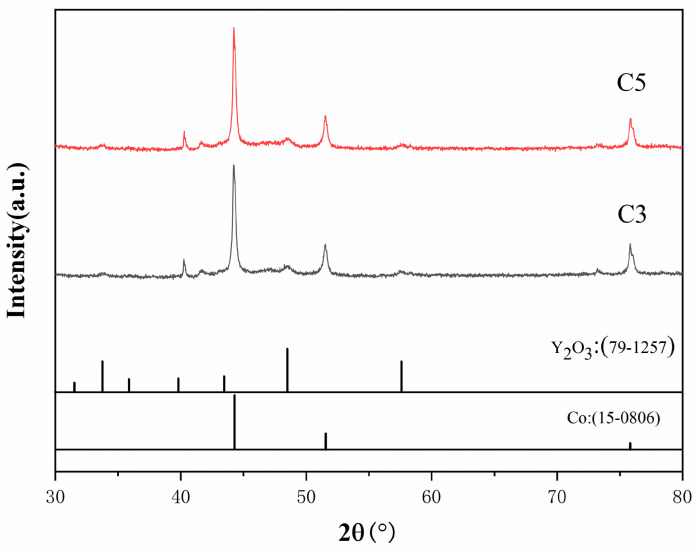
XRD patterns of Co composite powders with different Y contents.

The XRD outcomes of cobalt composite powders C3 and C5, with Y contents of 1.0% and 2.0%, respectively, following hydrogen reduction are presented in Figure 6. The reduction parameters are as follows: heating rate of 5 °C/min, reduction temperature of 600 °C, holding time of 60 min, and H_2_ flow rate of 1.0 m^3^/h. The reactions are as follows:

CoO + H_2_ = Co + H_2_O (g)

Co_2_O_3_ + 3H_2_ = 2Co + 3H_2_O (g)

Co_3_O_4_ + 4H_2_ = 3Co + 4H_2_O (g)

The (g) in the reaction equation indicates that the substances are in a gaseous state during the reaction.

As demonstrated in Figure 6, the peaks corresponding to the reduced Co phase and Y_2_O_3_ phase are clearly observed. It is noteworthy that no peaks corresponding to the Y_2_O_3_ phase were observed in the XRD pattern after calcination. This is because the formation of Y_2_O_3_ from Y(C_2_H_3_O_2_)_3_·4H_2_O occurs at temperatures above 400 °C, while the calcination temperature was only 300 °C, and the reduction temperature was 600 °C. Therefore, Y_2_O_3_ was formed during the reduction process. Furthermore, since the reduction temperature for Y_2_O_3_ is above 1000 °C, the 600 °C reduction temperature was insufficient to reduce the formed Y_2_O_3_. Consequently, the final oxide powder obtained after reduction was a Co-Y_2_O_3_ composite powder, as illustrated in Figure 6, which shows distinct peaks of Co and Y_2_O_3_. Additionally, it is evident that the XRD patterns for 1.0% and 2.0% rare earth Y content are almost identical.

**Figure 7:**
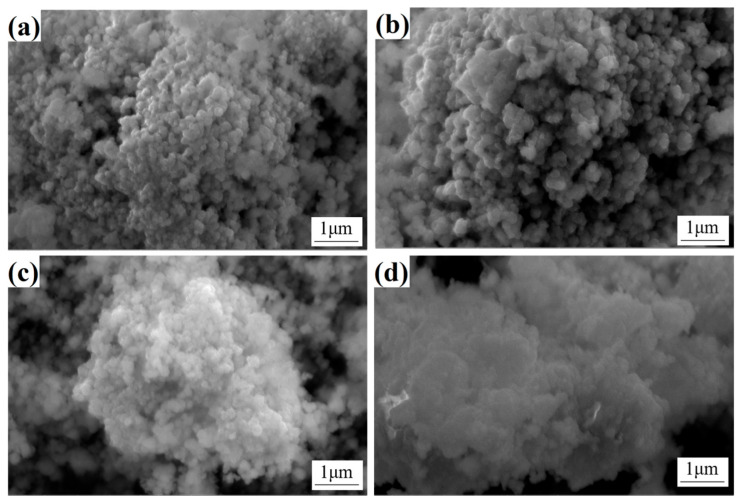
Microstructure of cobalt composite powders C1, C2, C3, and C5 (**a**) C1; (**b**) C2; (**c**) C3; (**d**) C5.

In order to facilitate a more profound comprehension of the raw material, SEM was employed to characterize the surface morphology of Co composite powders with varying Y content (see Figure 7). The Co powder particles exhibit uniform size, with an approximate diameter of 260 nm, and adhere to each other, forming clusters under the action of surface tension. The formation of these clusters can be attributed to the surface energy: following the processes of spraying, calcination and reduction treatment, the particle size of the Co powder is refined and uniform, and it possesses a substantial surface energy, which exerts a force on the powder particles to agglomerate, thereby reducing the total surface energy and thus reaching a state of equilibrium. The virtualization phenomenon on the surface of the powder is illustrated in Figure 7c,d and is attributed to the increased content of rare earth Y (which leads to a decrease in the conductivity of Co composite powder). In addition to being solidly soluble in the Co phase, Y also reacts with oxygen to form Y_2_O_3_, which has poor conductivity and is primarily distributed on the surface of the Co particles, thereby reducing the conductivity of the Co phase.

The CoO and Co_3_O_4_ powders containing the rare earth element Y were reduced to Co powder under hydrogen atmosphere at 600 °C. Concurrently, the high reduction temperature of Y_2_O_3_ means that the hydrogen atmosphere at 600 °C is unable to reduce Y_2_O_3_. Consequently, the powder becomes a Co-Y_2_O_3_ composite powder.

**Figure 8:**
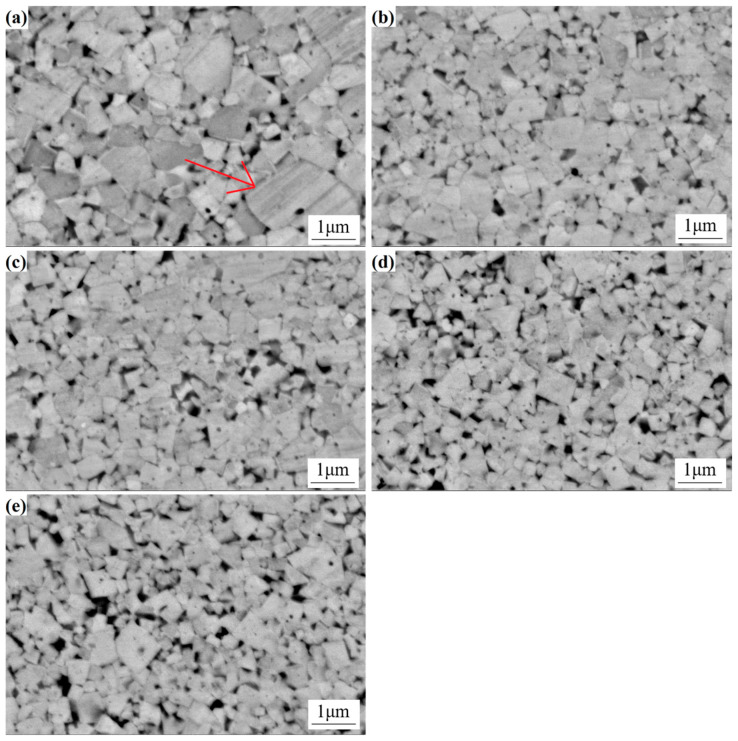
SEM figures of WC-6Co carbide with different Y element contents. (**a**) 0; (**b**) 0.5%; (**c**) 1.0%; (**d**) 1.5%; (**e**) 2.0%.

The microstructure of cemented carbide is a pivotal factor in alloy research as it dictates the material’s properties. A comprehensive investigation, utilizing SEM, was conducted to evaluate the microstructural evolution of sintered cemented carbide with varied Y content. The results, as shown in Figure 8, indicate significant differences in the microstructural characteristics among the different samples. In the absence of Y, the microstructure exhibits a broad WC grain size distribution, characterized by the prominence of larger grains (Figure 8a). Consequently, the average grain size attains a relatively large value of 0.76 µm. However, upon the introduction of Y, a marked transformation occurs, with WC grains becoming more uniform in size and distribution, narrowing the range, and reducing the average grain size. It is noteworthy that as the content of Y increases, a concomitant decrease in WC grain size is observed, which is indicative of the efficacy of Y in restraining the growth of WC grains within the cemented carbide matrix. This phenomenon underscores the potential of Y as a microstructural modifier, enhancing the properties of cemented carbide alloys.

In the cemented carbide devoid of the Y element, the grain size distribution range of WC is extensive, comprising a substantial number of substantial grains, with an average grain size of 0.76 µm. Subsequent addition of varying quantities of the Y element results in a narrowing of the grain size distribution range of WC, accompanied by a decrease in the average size. At a Y content of 1.5%, the average grain size of the alloy is recorded as 0.32 µm. This phenomenon can be attributed to the observation that the primary cause of WC grain growth is the dissolution of WC grains into the cobalt liquid phase during the liquid-phase sintering process, followed by their subsequent precipitation onto the surfaces of the inherent WC grains [24]. In this experiment, the spray drying method was employed, thereby enabling Y to initially dissolve into the Co phase during the spray drying process, which consequently hindered the dissolution of WC in Co during sintering. Furthermore, the uniform precipitation of Y from the cobalt phase during sintering results in its even distribution at the WC/Co interface. This effect exerts a pinning effect that hinders the grain boundary diffusion of WC grains and the dissolution-precipitation process of WC in the cobalt bonded phase, thereby suppressing the growth of WC grains [25].

As demonstrated in Figure 8, the absence of the Y element in cemented carbide results in a more dispersed distribution of WC grains, characterized by larger and more variable sizes. Conversely, as the content of the Y element increases, the WC grain size distribution becomes more uniform, exhibiting a narrower range and a smaller average grain size. This observation indicates that the Y element exerts a suppressive effect on WC grain growth, leading to a finer and more uniformly sized grain structure.

**Figure 9:**
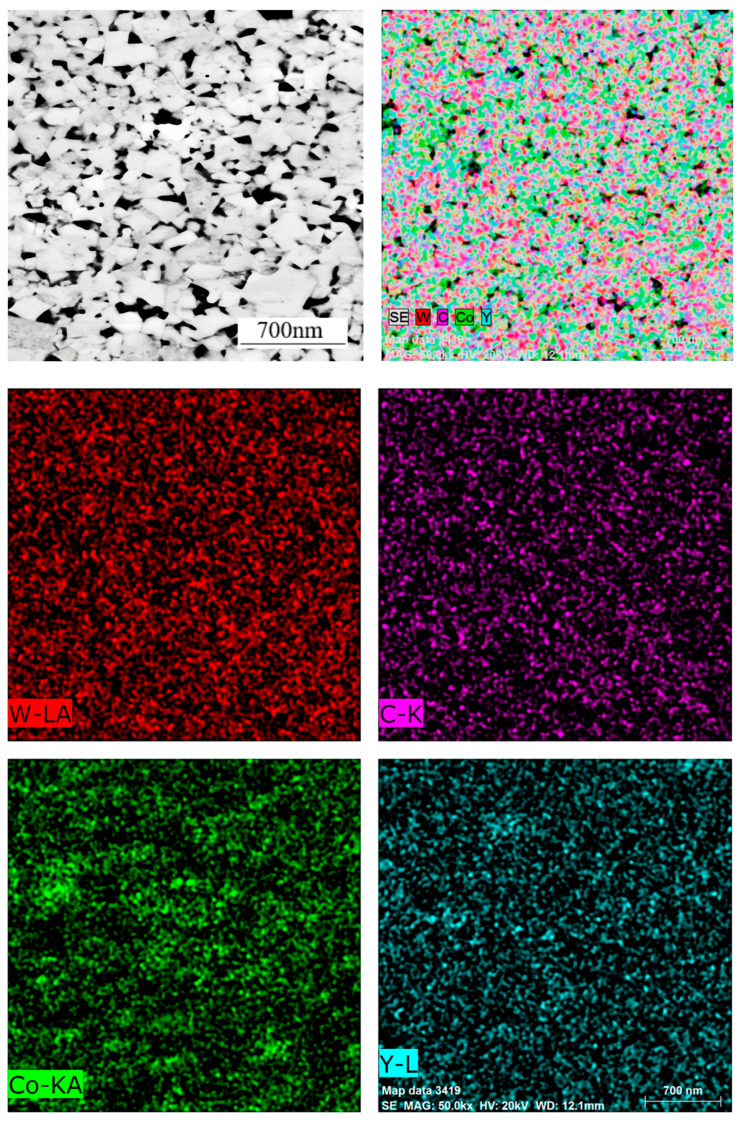

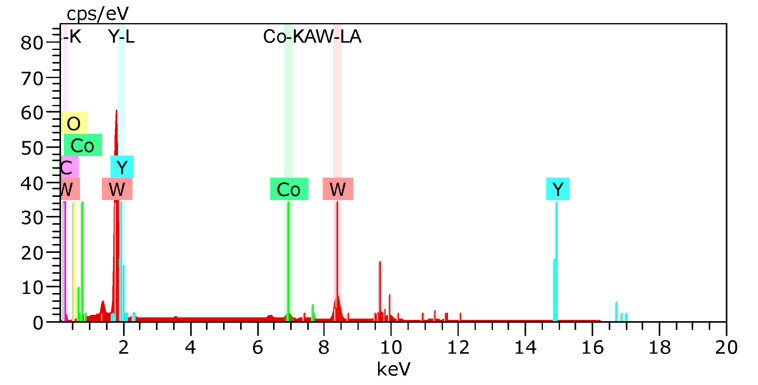
Elemental surface distribution of W, Co, and Y in Sample 4.

As illustrated in Figure 9, the distribution of element planes in sample No. 4 has been demonstrated. Surface scanning results indicate an absence of significant concentrations of the Co element. This phenomenon can be attributed to the utilization of Co powder with a particularly fine particle size, which has been shown to effectively mitigate or even eliminate the occurrence of a “cobalt pool” within the alloy.

**Figure 10:**
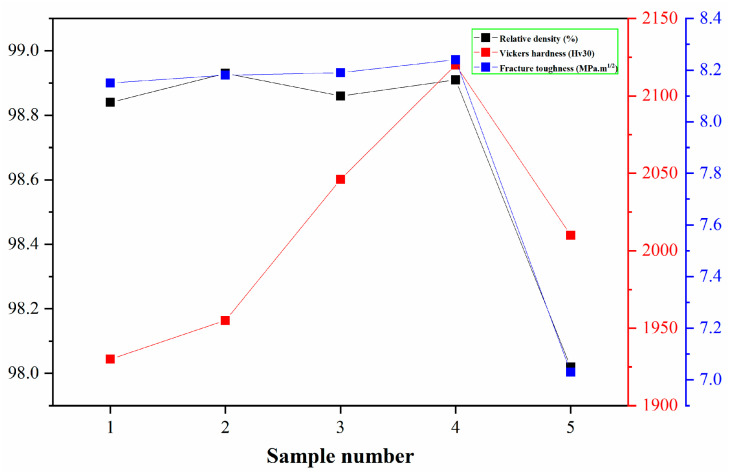
Curves of relative density, Vickers hardness, and fracture toughness of the cemented carbide.

As demonstrated in Table 3 and Figure 10, comparative data on the relative density, Vickers hardness, and fracture toughness of cemented carbides containing varying Y concentrations is presented. The initial four groups exhibit similar relative density values, all within an acceptable margin of error, suggesting that modest Y additions do not significantly impact the density of the alloys. However, upon reaching a Y content of 2%, a notable decrease in alloy density is observed. This decline is attributed to the pronounced precipitation of Y, which hinders the fluidity of the cobalt binder phase, thereby impeding its capacity to adequately fill the alloy’s internal porosity [20]. This finding underscores the critical role of Y content in modulating the microstructure and, consequently, the physical properties of cemented carbides. In a related study, Wu et al. [26] fabricated a Y-modified WC-12Co alloy and characterized its mechanical properties. Their findings indicated that the Y-doped cemented carbide exhibited a reduced relative density but augmented hardness and fracture toughness in comparison to the undoped alloy. This observation aligns with the results presented in this study, as illustrated in Table 3.

Hardness is a quantitative measure of a cemented carbide’s resistance to deformation under applied load, indicating the material’s inherent capacity to withstand indentation or scratching without undergoing plastic deformation. Greater porosity in the alloy corresponds to lower density, rendering the alloy more susceptible to deformation under compressive forces, which results in a decrease in hardness [28]. As demonstrated in Table 3, the presence of Y in amounts less than 0.5% results in a decline in hardness. The absence of Y leads to a hardness of 1930 Hv_30_, while the addition of 0.5% Y elevates the hardness to 1955 Hv_30_. The enhancement of hardness is associated with grain size: The addition of 0.5% Y has been shown to reduce WC grain size, and the hardness of the alloy increases with decreasing WC grain size in accordance with the Hall-Petch relationship [29]. As the Y content continues to increase, the hardness of the alloy rises to 2046 Hv_30_ and 2120 Hv_30_ at 1% and 1.5% Y content, respectively. However, when the Y content reaches 2%, although the WC grain size becomes smaller and the effect on improving the alloy value is more significant, the excessive Y content leads to the aggregation of some Y, reducing the fluidity of the cobalt bonding phase and hindering the filling of internal pores by cobalt [30]. The reduction in WC grain size leads to a proliferation of internal pores (evident in Figure 8e), ultimately causing a notable decline in the alloy’s hardness.

**Figure 11:**
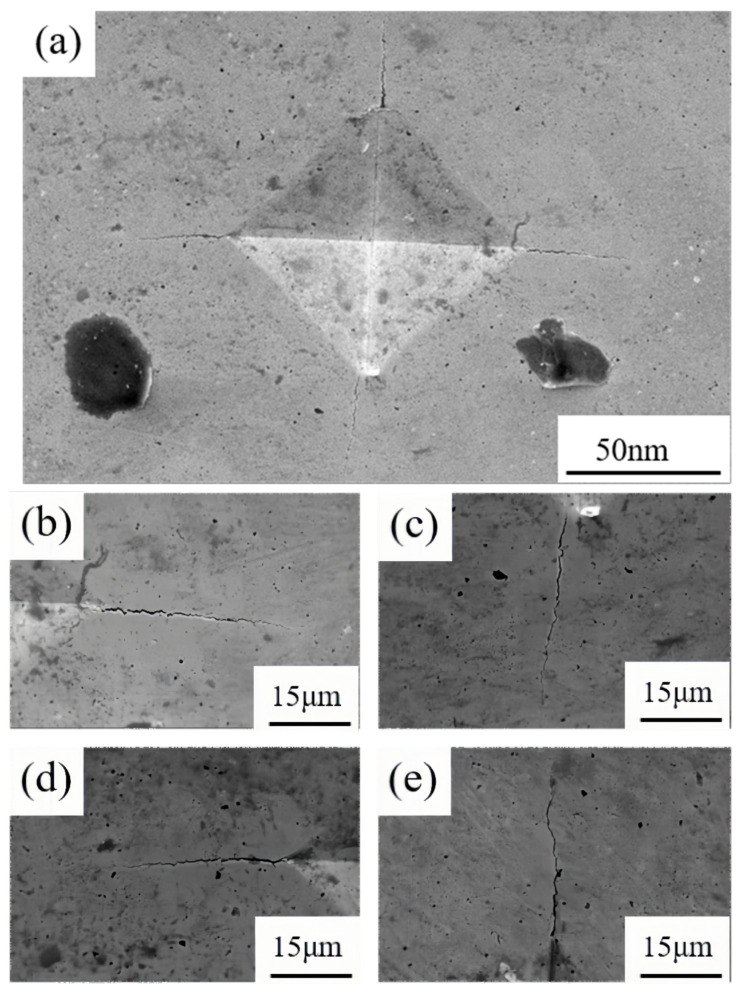
SEM image of the residual indentation on Sample 1: (**a**) the overall appearance of the indentation; (**b**) on the right; (**c**) below; (**d**) on the left; (**e**) on top.

Fracture toughness is a quantitative metric for the resistance of a cemented carbide to the extension of cracks within the material. The presence of pores in the alloy can be considered to have a detrimental effect on the fracture toughness of the material, as cracks can propagate more rapidly along the pore boundaries. As illustrated in Figure 11, which shows the SEM image of the residual indentation on Sample 1 and the crack situation at the four corners of the indentation, the fracture toughness of the alloy decreases with an increase in the number of pores. The results presented in Table 3 demonstrate that as the Y content is reduced below 1.5%, the alloy’s fracture toughness undergoes a gradual increase. However, when the Y content exceeds 2.0%, the toughness value undergoes a substantial decline, reaching a value of 7.03 MPa m^1/2^. As the Y content increases, the WC grain size decreases, thereby reducing the average free path of the Co phase in the alloy. This reduction in the average free path is the fundamental factor underlying the stability of fracture toughness. When cracks propagate, the average free path absorption deformation ability of the smaller Co phase weakens, resulting in a decrease in the fracture toughness of the alloy. Secondly, when the Y content reaches 2.0%, the density of the alloy significantly decreases, leaving more pores inside, making it easier for cracks to propagate, and also leading to a decrease in fracture toughness [31].

## 4. Conclusions

Through the implementation of innovative processes, namely spray drying, calcination, and reduction, the successful synthesis of Co-based composite powders containing rare earth Y was achieved. Utilizing Co-Y_2_O_3_ composite powder as a precursor, the preparation of WC-Co cemented carbides with varying Y content was accomplished through the integration of ball milling and spark plasma sintering. The outcomes of this study are as follows:

1. Following the calcination process, the samples are composed of the CoO and Co_3_O_4_ phases. It has been established that the CoO phase content in the samples increases in proportion to the increasing addition of rare earth Y, a factor that is beneficial for the subsequent reduction to Co-Y_2_O_3_ composite powder.
2. The addition of Y has been demonstrated to enhance the performance of the alloy; when the rare earth Y content is increased to 1.5%, the alloy exhibits good performance stability, with density and fracture toughness remaining unchanged, while Vickers hardness significantly increases. At a Y content of 1.5%, the alloy’s properties reach a state of stability, with a density of 98.91% and maximum values for hardness and fracture toughness of 2120 Hv_30_ and 8.24 MPa·m^1/2^, respectively. However, when the Y content is increased to 2.0%, the alloy’s density, fracture toughness, and hardness all decrease.
3. The present study investigates the dual action mechanism of rare earth Y in WC-Co carbide, with a view to highlighting the multifaceted influence of rare earth Y in WC-Co alloys. Incorporation of Y significantly aids in grain refinement and enhances the alloy’s hardness; however, excessive Y may cause phase change, lattice distortion, or an unfavorable phase, which may reduce the density and toughness of the alloy. Consequently, the meticulous regulation of Y content emerges as a pivotal strategy for harmonizing the alloy’s hardness, density, and resistance to fracture, ensuring optimal material properties.

## Figures and Tables

**Table 1:** Composition ratio of cemented carbide materials (wt.%).

Number	WC	Co	Y
Sample 1	94.0	6	0
Sample 2	93.5	6	0.5
Sample 3	93.0	6	1.0
Sample 4	92.5	6	1.5
Sample 5	92.0	6	2.0

**Table 2:** The physical phase content of CoO and Co_3_O_4_.

Number	CoO (wt.%)	Co_3_O_4_ (wt.%)
B1	40.6	59.4
B2	46.5	53.5
B3	47.5	52.5

**Table 3:** The relative density, Vickers hardness, and fracture toughness of the alloy.

Number	Relative Density (%)	Hv_30_	Fracture Toughness (MPa·m^1/2^)
Sample 1	98.84	1930	8.15
Sample 2	98.93	1955	8.18
Sample 3	98.86	2046	8.19
Sample 4	98.91	2120	8.24
Sample 5	98.02	2010	7.03
WC-12Co [27]	99.80	1417	10.90
WC-12Co-1.3Y_2_O_3_ [27]	99.70	1447	11.90

## Data Availability

The original contributions presented in this study are included in the article. Further inquiries can be directed to the corresponding authors.
